# The neuroanatomy of visual extinction following right hemisphere brain damage: Insights from multivariate and Bayesian lesion analyses in acute stroke

**DOI:** 10.1002/hbm.26639

**Published:** 2024-03-04

**Authors:** Christoph Sperber, Daniel Wiesen, Hans‐Otto Karnath, Bianca de Haan

**Affiliations:** ^1^ Center of Neurology, Division of Neuropsychology Hertie‐Institute for Clinical Brain Research, University of Tübingen Tübingen Germany; ^2^ Department of Neurology Inselspital, University Hospital Bern Bern Switzerland; ^3^ Department of Psychology University of South Carolina Columbia South Carolina USA; ^4^ Centre for Cognitive Neuroscience, College of Health and Life Sciences, Brunel University London Uxbridge UK

**Keywords:** intraparietal sulcus, selective attention, support vector regression, temporo‐parietal junction, visual extinction, VLSM

## Abstract

Multi‐target attention, that is, the ability to attend and respond to multiple visual targets presented simultaneously on the horizontal meridian across both visual fields, is essential for everyday real‐world behaviour. Given the close link between the neuropsychological deficit of extinction and attentional limits in healthy subjects, investigating the anatomy that underlies extinction is uniquely capable of providing important insights concerning the anatomy critical for normal multi‐target attention. Previous studies into the brain areas critical for multi‐target attention and its failure in extinction patients have, however, produced heterogeneous results. In the current study, we used multivariate and Bayesian lesion analysis approaches to investigate the anatomical substrate of visual extinction in a large sample of 108 acute right hemisphere stroke patients. The use of acute stroke patient data and multivariate/Bayesian lesion analysis approaches allowed us to address limitations associated with previous studies and so obtain a more complete picture of the functional network associated with visual extinction. Our results demonstrate that the right temporo‐parietal junction (TPJ) is critically associated with visual extinction. The Bayesian lesion analysis additionally implicated the right intraparietal sulcus (IPS), in line with the results of studies in neurologically healthy participants that highlighted the IPS as the area critical for multi‐target attention. Our findings resolve the seemingly conflicting previous findings, and emphasise the urgent need for further research to clarify the precise cognitive role of the right TPJ in multi‐target attention and its failure in extinction patients.

## Graphical abstract

Our results demonstrate that both the temporo‐parietal junction (TPJ) and the intraparietal sulcus (IPS) are critically associated with multi‐target attention and its failure in visual extinction. This resolves the seemingly conflicting previous findings where lesion studies have tended to implicate the TPJ, whereas studies in neurologically healthy participants have tended to implicate the IPS.

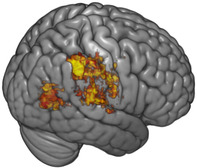

## 1 INTRODUCTION

Multi‐target attention, that is, the ability to attend and respond to multiple visual targets presented simultaneously on the horizontal meridian across both visual fields, is essential for everyday real‐world behaviour such as navigating traffic scenes, engaging in team sports, or playing a videogame. The importance of this ability is demonstrated particularly impressively in neurological patients suffering from extinction, typically as a consequence of right‐hemispheric brain damage (Becker & Karnath, 2007). These patients can report single unilateral visual targets in either visual field, but are unable to report the contralesional target in bilateral situations where an ipsilesional target is concurrently present (de Haan et al., 2012; Oppenheim, 1885). Extinction is most commonly seen as a consequence of biased competitive interactions between the ipsilesional and contralesional target stimuli, and an exaggeration of the difficulty that healthy subjects have while trying to attend and respond to multiple targets presented simultaneously (de Haan et al., 2012; Desimone & Duncan, 1995; Driver et al., 1997; Duncan, 1998; Duncan et al., 1997; Mattingley, 2002). Given this close link between extinction and attentional limits in healthy subjects, investigating the anatomy that underlies extinction is uniquely capable of providing important insights concerning the anatomy critical for normal multi‐target attention.

Early studies in acute stroke patients have implicated the right temporo‐parietal junction (TPJ) in visual extinction (Karnath et al., 2003; Ticini et al., 2010). These studies performed descriptive lesion/malperfusion subtraction analyses, that allow us to determine which areas of the brain are more frequently damaged or malperfused in patients with than in patients without extinction (Rorden & Karnath, 2004), but allow no statistical inference. Other lesion studies statistically assessed the relationship between visual extinction severity and lesion location (Beume et al., 2020; Chechlacz, Rotshtein, et al., 2013; Chechlacz, Terry, et al., 2013; Hillis et al., 2006; Vossel et al., 2011). These studies have, however, produced heterogeneous findings. In line with the findings from the descriptive lesion studies, Chechlacz, Rotshtein, et al. (2013) and Chechlacz, Terry, et al. (2013) (Analysis 1) found that visual extinction was associated with damage centring on the right TPJ. Other results have, however, implicated the right angular gyrus (Chechlacz, Rotshtein, et al., 2013, Analysis 2 and 3; Vossel et al., 2011), the right supramarginal gyrus, medial temporal gyrus, and medial frontal gyrus (Chechlacz, Terry, et al., 2013), the right inferior occipital gyrus (Hillis et al., 2006), or the left intraparietal sulcus (IPS), inferior parietal lobe, and supramarginal gyrus (Beume et al., 2020) in visual extinction. This heterogeneity of findings is echoed in neurodisruption studies in neurologically healthy participants. In line with the early descriptive lesion studies, Meister et al. (2006) found that a temporary disruption of neural activity at the right TPJ resulted in extinction‐like behaviour. Other neurodisruption studies, however, report extinction‐like behaviour following a temporary disruption of neural activity at the right (Cazzoli et al., 2009; Hung et al., 2005; Koch et al., 2005), or either the left or right IPS (Battelli et al., 2009; Hilgetag et al., 2001; Pascual‐Leone et al., 1994).

Part of this heterogeneity across studies concerning the area(s) of the brain implicated in multi‐target attention and its failure in visual extinction may be due to subtle differences in methodology and analysis approach between studies (e.g. which covariates to include in the statistical lesion analysis, which brain area to target when applying transcranial magnetic stimulation). However, this heterogeneity in previous analysis results may also reflect a more fundamental issue in these studies: the potential underestimation of the full extent of areas of the brain critical for multi‐target attention and its failure in extinction patients.

Firstly, neurodisruption studies in neurologically healthy participants by definition focus on a small area of the brain. As such, while these studies have been hugely informative when it comes to understanding the contribution of the area of the brain targeted to a cognitive function of interest, they provide little information about the areas of the brain not targeted. More fundamentally, neurodisruption studies require advance knowledge of the area of the brain implicated in the cognitive function of interest. As such, while neurodisruption techniques are excellently suited to precisely study the function of the targeted brain area, they are less suitable for the detection of the full extent of areas of the brain implicated in a cognitive function of interest (Walsh & Rushworth, 1999).

Second, the vast majority of previous statistical lesion analysis studies investigating the anatomy underlying visual extinction relied on non‐acute patient data. Time since stroke is an important factor to consider in statistical lesion analysis studies, and each time point from the acute to the chronic stage has benefits and limitations. A limitation associated with the use of non‐acute stroke patient data is that results may be affected by functional reorganisation of the brain in the course of normal recovery (de Haan & Karnath, 2018; Karnath & Rennig, 2016; Karnath & Rorden, 2012). When a lesion analysis is performed using non‐acute stroke patient data, the parts of the brain damaged in patients who have fully or partially recovered from their initial deficit are erroneously assumed to be not, or less critically, associated with the cognitive function of interest. As a consequence, the lesion analysis may fail to fully identify all areas of the brain associated with this cognitive function of interest.

Finally, all lesion studies conducted so far to statistically assess the anatomical substrate of visual extinction used a univariate and frequentist lesion analysis approach. Such univariate lesion analysis approaches have limitations. Firstly, in univariate lesion analysis approaches, each voxel is considered an independent contributor to behaviour. Brain functions are, however, not organised in single voxels, but instead in larger functional areas or networks (Mah et al., 2014; Pustina et al., 2018). Univariate approaches are computationally limited in their ability to detect these larger functional networks. Additionally, univariate lesion analysis approaches are vulnerable to the so‐called “partial injury problem” (Rorden et al., 2009; Sperber, Wiesen, & Karnath, 2019). This problem occurs when the behavioural deficit of interest is seen following non‐overlapping damage to different parts of the same functional area or network in different patients. In this situation, a univariate approach may, again, not be able to detect the full extent of the areas of the brain associated with the cognitive function of interest. Multivariate lesion analysis approaches, which simultaneously consider the contribution of multiple voxels to behaviour, may be more appropriate (Karnath et al., 2018; Mah et al., 2014; Pustina et al., 2018). Several simulation studies have shown that multivariate lesion analysis approaches can indeed be superior to univariate lesion analysis approaches in detecting brain networks (Mah et al., 2014; Pustina et al., 2018; Zhang et al., 2014). Second, all previous statistical lesion analysis studies used frequentist statistics. Frequentist statistics, however, cannot provide evidence for the null hypothesis (i.e., show that a brain region is *not* the neural correlate of a function). As a result, previous studies that identified different neural correlates of visual extinction may not, strictly speaking, contradict each other. For example, a frequentist lesion analysis that only implicates the supramarginal gyrus does not provide information about the role of other areas—the TPJ may or may not be relevant for extinction, we simply do not know. This limitation can be overcome by Bayesian lesion‐deficit inference (BLDI), which allows us to assess evidence for the null hypothesis, as well as detect voxels with insufficient evidence for either the null or the alternative hypothesis (Sperber et al., 2023). Importantly, BLDI performs better when statistical power is low (Sperber et al., 2023). As such, this approach may be better suited to detect functional contributions in areas of the brain less frequently damaged by stroke lesions, such as, for example, the superior parietal lobe and IPS, allowing us, again, to obtain a fuller picture of the areas of the brain associated with the cognitive function of interest.

In the current study, we use multivariate and Bayesian statistical lesion analysis approaches to investigate the anatomical substrate of visual extinction in a large sample of 108 acute right hemisphere stroke patients. The use of acute stroke patient data together with multivariate and Bayesian lesion analysis approaches addresses the limitations associated with previous studies and so potentially allows us to gain a more complete picture of the brain areas critical for multi‐target attention and its failure in extinction patients.

## 2 METHODS AND MATERIALS

### 2.1 Patients

We, retrospectively, analysed data of 108 patients who had been admitted to the Tübinger Center of Neurology with a first‐ever right hemisphere unilateral stroke (see Table 1 for clinical and demographic data). Like neglect, extinction is far less common after left than after right brain damage (Becker & Karnath, 2007). As a result, obtaining sample sizes sufficiently large to conduct a multivariate statistical lesion analysis is far more feasible in right brain damaged patients than in left brain damaged patients. Inclusion criteria were: no evidence of older infarcts, no diffuse, bilateral, or cerebellar lesions, and no evidence of other neurological or psychiatric disorders. Neuropsychological assessment and imaging were performed as part of the clinical protocols during acute inpatient care at the stroke unit. Patients or their relatives consented to the scientific re‐use of their data. The study was performed in accordance with the ethical standards laid down in the 2013 Declaration of Helsinki.

**TABLE 1:** Clinical and demographic data of all patients, and for patients with at least one contralesional omission during bilateral trials in the clinical confrontation assessment for visual extinction versus patients without any omissions. All numbers are reported as mean (standard deviation; minimum; maximum), except visual field defects, for which the number of patients without field defects/ with quadrantanopia/ with hemianopia are reported, and sex.

	All (*N* = 108)	Patients with ≥1 contralesional omission (*N* = 42)	Patients without any contralesional omissions (*N* = 66)
Extinction score (omissions during bilateral presentation) (%)	25.3 (38.9; 0; 100)	65.0 (36.1; 10; 100)	0 (0; 0; 0)
Spatial neglect (CoC score)	0.13 (0.21; −0.04; 0.85)	0.23 (0.26; −0.04; 0.80)	0.07 (0.14; −0.04; 0.85)
Age (years)	59.6 (13.3; 27; 93)	58.8 (13.1; 27; 80)	60.2 (13.5; 30; 93)
Sex (F, M)	50/58	18/24	32/24
Time lesion to scan (days)	2.2 (2.3; 0; 8)	2.7 (2.3; 0; 8)	1.9 (2.4; 0; 8)
Time lesion to assessment (days)	2.9 (1.9; 0; 7)	2.7 (1.9; 0; 7)	3.1 (1.9; 0; 7)
Lesion size on normalised scan (cm^3^)	39.8 (41.5; 0.5; 234.8)	62.4 (50.4, 1.4; 234.8)	25.4 (26.2; 0.5; 103.4)
Visual field defects (no/QA/HA)	100/3/5	38/1/3	62/2/2

### 2.2 Neuropsychological assessment

Patients were neuropsychologically assessed in the acute post‐stroke stage 2.9 days (SD = 1.9; range 0–7 days) after stroke onset (see Table 1). The neurological assessment protocol consisted of assessments for visual field defects, spatial neglect, and visual extinction.

Visual field defects were assessed with the clinical confrontation technique, where the patient was required to detect a movement of the examiner's left or right index finger, presented in the patient's left or right visual field. Each patient was presented with six movements in each visual field, two in the upper quadrant, two on the horizontal meridian, and two in the lower quadrant.

Spatial neglect was assessed with two cancellation tasks. Each cancellation task was administered as a paper and pencil test on a 21.0 cm × 29.7 cm A4 sheet of paper, placed in landscape orientation on the patient's sagittal midline. Patients were instructed to manually cancel out certain target items that were presented in a larger array of items including both target and distractor items. In the bells cancellation task (Gauthier et al., 1989), 35 solid black objects in the shape of bells had to be found among other black solid distractor items. In the letter cancellation task (Weintraub & Mesulam, 1985), 60 letters “A” had to be found among other letters. No time limit was set for completion. The cancellation performance was evaluated by calculating the Center of Cancellation (CoC), a continuous measure that assesses the egocentric core component of spatial neglect (Rorden & Karnath, 2010). The CoC scores of both tests were averaged to obtain a single score. A CoC of 0 indicates a symmetrical cancellation performance; CoCs above 0 indicate a neglect‐typical right‐ward shift, with a CoC of 1 indicating maximal possible neglect.

Visual extinction was assessed with a variation of the clinical confrontation technique where the patient was required to detect a movement of the examiner's left and/or right index finger presented in the patient's left and/or right visual field. Each patient was presented with 10 unilateral left, 10 unilateral right, and 10 bilateral movements. If a patient displayed a visual field defect, care was taken to present the movements in the intact part of the visual field: In patients with lower or upper left visual field quadrantanopia (*n* = 3), movements were presented in the intact upper or lower visual field respectively. In patients with left visual field hemianopia (*n* = 5), movements were presented in the near and/or far periphery of the intact ipsilesional visual field. An extinction score was calculated as the percentage of bilateral trials in which the patient failed to detect the contralesional movement. The percentage of correct detection of contralesional movements during unilateral stimulation and ipsilesional movements was not recorded during the neuropsychological assessment for visual extinction. However, the extinction assessment protocol stipulated that any patient who correctly detected less than 90% of the contralesional movements during unilateral stimulation was classified as “not assessable,” and these patients were excluded from the current study. Moreover, trials in which the patient did not respond to the ipsilesional movement were excluded, and in those instances a check was performed to ensure the patient understood the task. As such, all patients included in the current study correctly detected at least 90% of the contralesional movements during unilateral stimulation, and correctly detected all ipsilesional movements.

For a subset of 39 patients without visual field defects (mean age = 56.7 years, SD = 11.2; 15 females, mean time between stroke and testing 3.4 days, SD = 2.1, range 0–7 days), visual extinction was additionally assessed using a computerised test with time‐critical target presentation that allowed measurement of performance during both unilateral and bilateral trials, and the calculation of a so‐called “extinction index” (Vossel et al., 2011). Each trial started with a central white fixation cross (0.6° × 0.6° visual angle) presented on a black background for a duration of 500 ms. Patients were instructed to continuously fixate this fixation cross. This was followed by the presentation of a peripheral white target stimulus on the horizontal midline at an eccentricity of 10.0° visual angle for a duration of 180 ms. The target stimulus was a white geometrical shape (circle, square, triangle, or diamond; 1.5° × 1.5° visual angle), that was presented either unilaterally left, unilaterally right, or bilaterally. During bilateral presentations, the target stimuli were never identical. Patients were required to vocally report the location and shape of the target(s) presented (i.e. “circle left” or “diamond left and triangle right”) while the neuropsychologist logged these vocal responses on a sheet of paper. Finally, after the neuropsychologist had made sure the patient was fixating the central fixation cross; the next trial was initiated by the neuropsychologist with a keyboard response. In a single session, patients were presented with 10 unilateral left, 10 unilateral right and 10 bilateral targets in a pseudo‐randomised order that was fixed over patients. The proportion correct (ranging from 0 to 1) during unilateral left and right target presentations, and bilateral target presentations was used to calculate an extinction index according to the following formula I_ext_ = (P_(hit|uni‐left)_‐P_(hit|bil‐left)_)–(P_(hit|uni‐right)_‐P_(hit|bil‐right)_) (taken from Vossel et al., 2011). This extinction index ranges from 1 to −1 with an index of 1 reflecting complete contralateral extinction and an index of −1 reflecting complete ipsilateral extinction.

To achieve sufficient statistical power for the multivariate statistical lesion analysis (Sperber, Wiesen, & Karnath, 2019), we used the extinction score as determined using the clinical confrontation technique described above, as all 108 patients were assessed for extinction using this approach, meaning the entire sample could be included in the analysis. The extinction score, as obtained using the clinical confrontation technique, was controlled for potential effects of unilateral attentional biases on performance by regressing out the variance explained by spatial neglect from the extinction score via nuisance regression. Neglect impacts contralesional performance during both unilateral and bilateral trials, whereas extinction impacts contralesional performance solely during bilateral trials. As such, regressing out the variance explained by neglect from the extinction score should be similar to the use of an extinction index as documented in the previous literature (Chechlacz, Rotshtein, et al., 2013; Chechlacz, Terry, et al., 2013; Chechlacz et al., 2014; Vossel et al., 2011), where contralesional attentional biases are controlled for by calculating the difference in performance during unilateral and bilateral trials. Specifically, we used linear regression to regress the extinction score as assessed using the clinical confrontation technique on the neglect score, that is, the average CoC. The residuals of this linear regression provide a measure of extinction that is controlled for the potential effects of unilateral attentional biases. In the multivariate statistical lesion analysis, we used these residuals as our behavioural variable of interest.

### 2.3 Validation of covariate control approach

Controlling for behavioural covariates is not always appropriate in lesion analyses, and can even be detrimental (Sperber et al., 2020). An exception to this, however, is the case where the behavioural covariate directly influences the variable of interest. Unilateral attentional deficits such as spatial neglect directly affect our measure of visual extinction as assessed by omissions during bilateral trials in the clinical confrontation technique. As such, these unilateral attention deficits constitute an actual confound that should be controlled for (see Sperber et al., 2020). To assess whether our approach of regressing out the variance explained by spatial neglect using nuisance regression is an effective control for the potential effects of unilateral attentional biases on performance in the visual extinction assessment, we conducted a validation. This validation used the subset of 39 patients without visual field defects, who were assessed on spatial neglect, as well as on visual extinction using a computerised test with time‐critical target presentation that allowed us to measure performance during both unilateral and bilateral trials. We used the performance during unilateral and bilateral trials in the computerised test to calculate an extinction index as in Vossel et al. (2011). This provides a measure of extinction while controlling for unilateral biases. Additionally, we used the performance during bilateral trials in the computerised test to obtain an extinction score. As in the assessment using the clinical confrontation technique, this extinction score reflected the percentage of bilateral trials in which the patient failed to detect the contralesional target.

As in the multivariate statistical lesion analysis, we used linear regression to regress the extinction score, that is, the percentage contralesional omissions during bilateral trials, on the neglect score, that is, the average CoC. Subsequently, we performed a Spearman's rank order correlation analysis to assess the correlation between the residuals of the linear regression and the extinction index calculated as in Vossel et al. (2011). If controlling for the potential effects of unilateral attentional biases on performance in the visual extinction assessment using nuisance regression is effective, we would expect a strong and significant correlation between the residuals of this linear regression and the extinction index.

### 2.4 Imaging and lesion mapping

**FIGURE 1:**
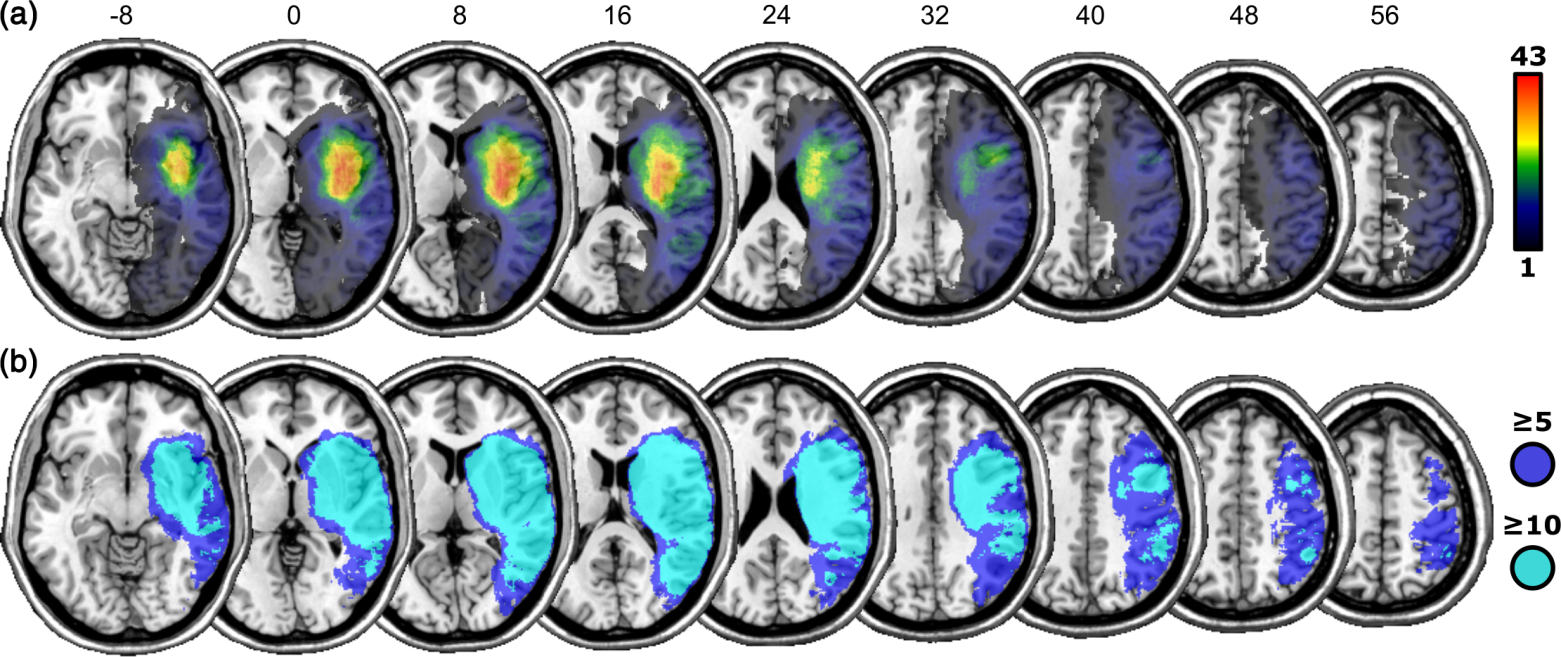
(a) Lesion overlap map of all 108 lesions. (b) Areas of the brain assessed in the multivariate lesion analysis (areas damaged in at least 10 patients), and in the Bayesian analysis (areas damaged in at least five patients). Numbers above slices indicate z‐coordinates in MNI‐space.

Brain imaging was obtained in the acute post‐stroke stage, on average 2.2 days (SD = 2.3; maximum 8 days) after stroke onset (see Table 1). Acute clinical imaging was obtained from all patients either by CT or MR. If adequate imaging of both imaging modalities was available, MR was preferred. Only scans that displayed clearly demarcated lesions were used. For patients with MR, diffusion‐weighted imaging was used in the hyperacute stage up to 48 h after stroke onset, and T2‐weighted fluid‐attenuated inversion recovery imaging afterwards (de Haan & Karnath, 2018). Lesions were manually drawn on transversal slices of the clinical scan using MRIcron (https://www.nitrc.org/projects/mricron). Lesion delineation was performed by experienced researchers (BdH, CS, and DW) and verified by consensus with a neurologist with longstanding expertise in lesion mapping (HOK). Normalisation of individual lesion maps to a common space was performed by Clinical Toolbox (Rorden et al., 2012). This software contains age‐specific normalisation templates both for CT and MR imaging. The lesioned area was controlled for in the normalisation either using cost‐function masking or enantiomorphic normalisation. A lesion overlap map can be seen in Figure 1.

### 2.5 Support vector regression‐based lesion‐symptom mapping

The multivariate statistical lesion analysis was performed with support vector regression‐based lesion‐symptom mapping (SVR‐LSM; DeMarco & Turkeltaub, 2018; Sperber, Wiesen, & Karnath, 2019; Zhang et al., 2014). Contrary to univariate methods, this multivariate approach to lesion behaviour mapping models the lesion status of all voxels in the brain at once.

We used SVR (Drucker et al., 1996), which is a machine learning‐based multivariate regression approach. All voxels damaged in at least 10 patients were included in this analysis. This threshold was chosen to ensure sufficient statistical power at each voxel for the analysis (Sperber & Karnath, 2022). The downside, however, is that areas of the brain rarely damaged were excluded. The damage status of each voxel was used to model the behavioural outcome variable, that is, the continuous extinction scores controlled for spatial neglect. Following the procedures in previous studies (DeMarco & Turkeltaub, 2018; Sperber, Wiesen, Goldenberg, & Karnath, 2019; Sperber, Wiesen, & Karnath, 2019; Wiesen et al., 2019; Zhang et al., 2014), we used a non‐linear ε‐SVR with radial basis function kernel. A control for lesion size was implemented by direct total lesion volume control (Zhang et al., 2014). We computed voxel‐wise feature weights, so‐called pseudo β‐parameters, based on approximations for the non‐linear kernel (see appendix in Zhang et al., 2014). Hyperparameters *C* and *γ* were optimised by a fivefold cross‐validation grid search. For this fivefold procedure, prediction accuracy and reproducibility of β‐parameters were assessed (Zhang et al., 2014). To assess prediction accuracy, the SVR model taken from four‐fifths of the data was used five times to predict data in the last fifth of the data. Then, the average correlation between real and predicted scores out of all five runs was assessed. To assess the reproducibility of β‐parameters, the SVR model was five times computed for four‐fifths of the data. Reproducibility was subsequently assessed by computing the average correlation of β‐parameters between all these subsets. We aimed to find hyperparameters that provided high reproducibility while still providing decent prediction accuracy (Rasmussen et al., 2012; Zhang et al., 2014). Considering that Zhang et al. (2014) performed a coarse grid search that pointed at a smaller set of viable hyperparameters, we only performed a fine grid search in the suggested range of *C* = [1, 10, 20, 30, 40, 50, 60, 70, 80] and *γ* = [0.1, 1, 2, 3, 4, 5, 6, 7, 8, 9, 10, 15, 20, 25, 30]. Voxel‐wise statistical inference was computed by permutation testing the β‐parameters obtained in the SVR (Zhang et al., 2014) with 10,000 permutations, and *p*‐values were remapped into brain space. We used a false discovery rate (FDR) at *q* = 0.1 to correct for multiple comparisons. Statistical maps were anatomically interpreted with the aid of brain atlases. Clusters of significant voxels in grey matter areas were anatomically interpreted using the maximum probability map of the Loni Probabilistic Brain Atlas (Shattuck et al., 2008), and clusters of significant voxels in white matter areas were interpreted using maps of long association fibres in a probabilistic white matter atlas (Zhang et al., 2010). The probabilistic white matter maps were thresholded at *p* ≥ .4 to obtain a binary map for each fibre tract. All analyses were done using MATLAB 2018 and libSVM 3.21. The SVR‐LSM analysis was performed using custom modified scripts based on the scripts by Zhang et al. (2014) (https://github.com/yongsheng-zhang/SVR-LSM).

### 2.6 Bayesian lesion‐deficit inference

BLDI by Bayes factor mapping was performed with the BLDI toolkit (Sperber et al., 2023) in R. BLDI is a univariate approach that weighs for each imaging voxel the evidence for the alternative hypothesis H1 that a lesion‐deficit association exists against the null hypothesis H0 that no such association exists. Contrary to common frequentist methods, BLDI can provide evidence for H0, and transparently highlights brain areas for which no evidence for either hypothesis exists, such as voxels for which data provide insufficient statistical power to gauge the evidence. We applied Bayesian general linear models in each voxel lesioned in at least five patients. We chose a lower threshold than for the SVR‐LSM described above to include additional areas of the brain that were less frequently damaged, as BLDI has previously been shown to be well‐suited to detect functional contributions of less‐frequently damaged areas of the brain, that is, where statistical power is low (Sperber et al., 2023). We used the extinction score as determined using the clinical confrontation technique as predictor and the average CoC score as a covariate, to map Bayes factors across the brain. The Bayes factor is a ratio that indicates the probability of H1 against H0. For example, a Bayes factor of 15 indicates that H1 is 15 times more likely than H0 and a Bayes factor of 1/10 indicates that H0 is 10 times more likely than H1. We interpreted Bayes factors in line with established standards (Wagenmakers et al., 2018), suggesting Bayes factors >3 as evidence for H1 and Bayes factors <1/3 as evidence for H0.

For a closer examination of the role of the IPS, we obtained probabilistic maps from the SPM Anatomy Toolbox v2.0 (Eickhoff et al., 2005). We summed the three maps of the IPS subparts and reoriented them into the imaging space of our analysis. We investigated the Bayes factors once for the maximum extent of the probabilistic map (*p* > 0) and once for the core area (*p* ≥ .4).

## 3 RESULTS

### 3.1 Validation of covariate control approach

In the sub‐sample of 39 patients where visual extinction was assessed with the computerised visual extinction task, on average 33% (SD = 38) of left stimuli in bilateral trials were omitted, and the extinction index was on average .25 (SD = .34). As expected, the results of our linear regression revealed that spatial neglect was a significant predictor of the extinction score (*β* = 1.199, *p* < .0001, *R* ^2^ = .467). More importantly, the Spearman's rank order correlation analysis revealed a strong and significant positive correlation between the residuals of the linear regression and the extinction index (*ρ* = .737, *p* < .0001). This suggests our method of controlling for potential effects of unilateral attentional biases on performance in the visual extinction assessment using nuisance regression is effective.

### 3.2 SVR‐based LSM

The average size of the brain lesion in our stroke patients was 39.8 cm^3^ (SD = 41.5). The average extinction score, as obtained using the clinical confrontation technique, was 25% (SD = 39). Then, 42 out of the 108 patients omitted at least one left‐sided stimulus in bilateral trials (see Table 1 for detailed demographical and clinical data).

**FIGURE 2:**
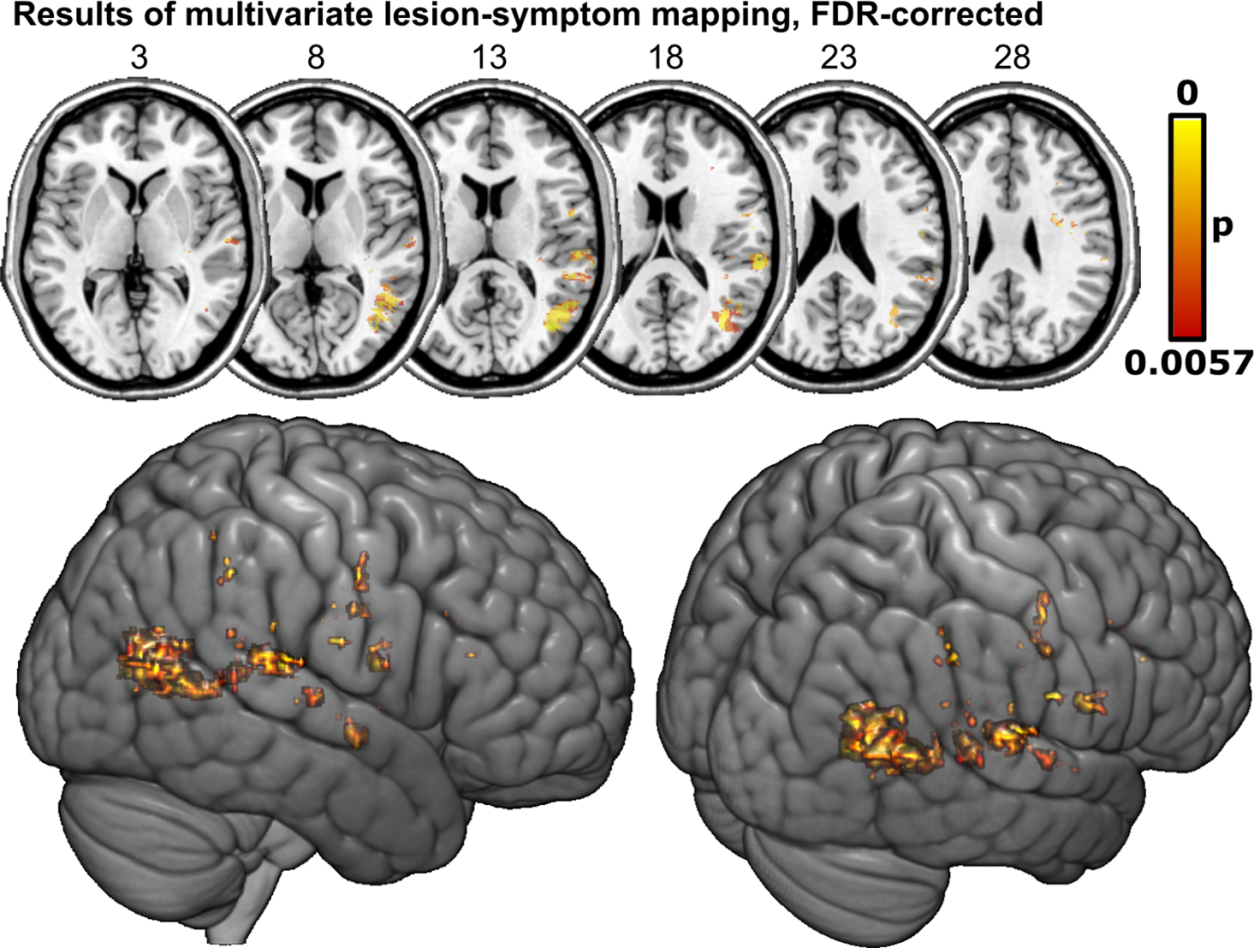
Neural substrates of visual extinction in acute stroke mapped by support vector regression‐based lesion‐symptom mapping (SVR‐LSM). Extinction scores underlying the topography were controlled for spatial neglect. Permutation‐thresholded voxel‐wise results of SVR‐LSM with false discovery rate (FDR) correction at *q* = 0.1.

The grid search revealed the hyperparameters *C* = 40 and *γ* = 2 to be optimal with a prediction accuracy of *r* = .38 and a reproducibility of *r* = .88. The SVR‐LSM identified 6700 suprathreshold voxels at an FDR of *q* = 0.1, equivalent to *p* < .0057 (see Figure 2 and Table 2). Most significant voxels were found in three larger clusters in inferior occipito‐temporo‐parietal regions in and around the TPJ. The largest cluster, abutting the infero‐posterior end of the TPJ, included the middle occipital gyrus, angular gyrus, and posterior parts of the middle temporal gyrus. Two other larger clusters in the TPJ were found in the posterior superior temporal gyrus and inferior supramarginal gyrus. Significant voxels were nearly exclusively found in grey matter areas, except for a few voxels (approx. 5% of all suprathreshold voxels) reaching into parts of the superior longitudinal fasciculus.

**TABLE 2:** Localisation of clusters of >100 significant voxels in the SVR‐LSM analysis after FDR correction at *q* = 0.1 as assigned by the Loni Probabilistic Brain Atlas (Shattuck et al., 2008) and a probabilistic white matter atlas (Zhang et al., 2010) for long association fibres. Only regions with >15 voxels assigned to a cluster are reported. All numbers are in mm^3^.

Cluster number	1	2	3	4	5	6	7	8
Total cluster size	3963	781	329	196	181	180	173	114
*Grey matter regions* (*Loni atlas*)								
Middle occipital gyrus	2307	0	0	0	0	0	0	0
Middle temporal gyrus	1048	0	4	0	0	0	0	0
Superior temporal gyrus	7	524	315	196	0	0	0	0
Angular gyrus	572	0	10	0	0	0	0	73
Precentral gyrus	0	0	0	0	158	180	0	0
Supramarginal gyrus	0	257	0	0	0	0	0	41
Hippocampus	0	0	0	0	0	0	69	0
*White matter regions* (*white matter atlas*)								
SLF—parieto‐temporal	284	0	0	0	0	0	0	0
SLF—fronto‐parietal	0	0	0	0	0	83	0	0

*Note*: Our results demonstrate that both the TPJ and the IPS are critically associated with multi‐target attention and its failure in visual extinction. This resolves the conflicting previous findings where lesion studies have tended to implicate the TPJ, whereas studies in neurologically healthy participants have tended to implicate the IPS.

Abbreviations: FDR, false discovery rate; IPS, intraparietal sulcus; SLF, superior longitudinal fasciculus; SVR‐LSM, support vector regression‐based lesion‐symptom mapping; TPJ, temporo‐parietal junction.

### 3.3 Bayesian lesion‐deficit inference

**FIGURE 3:**
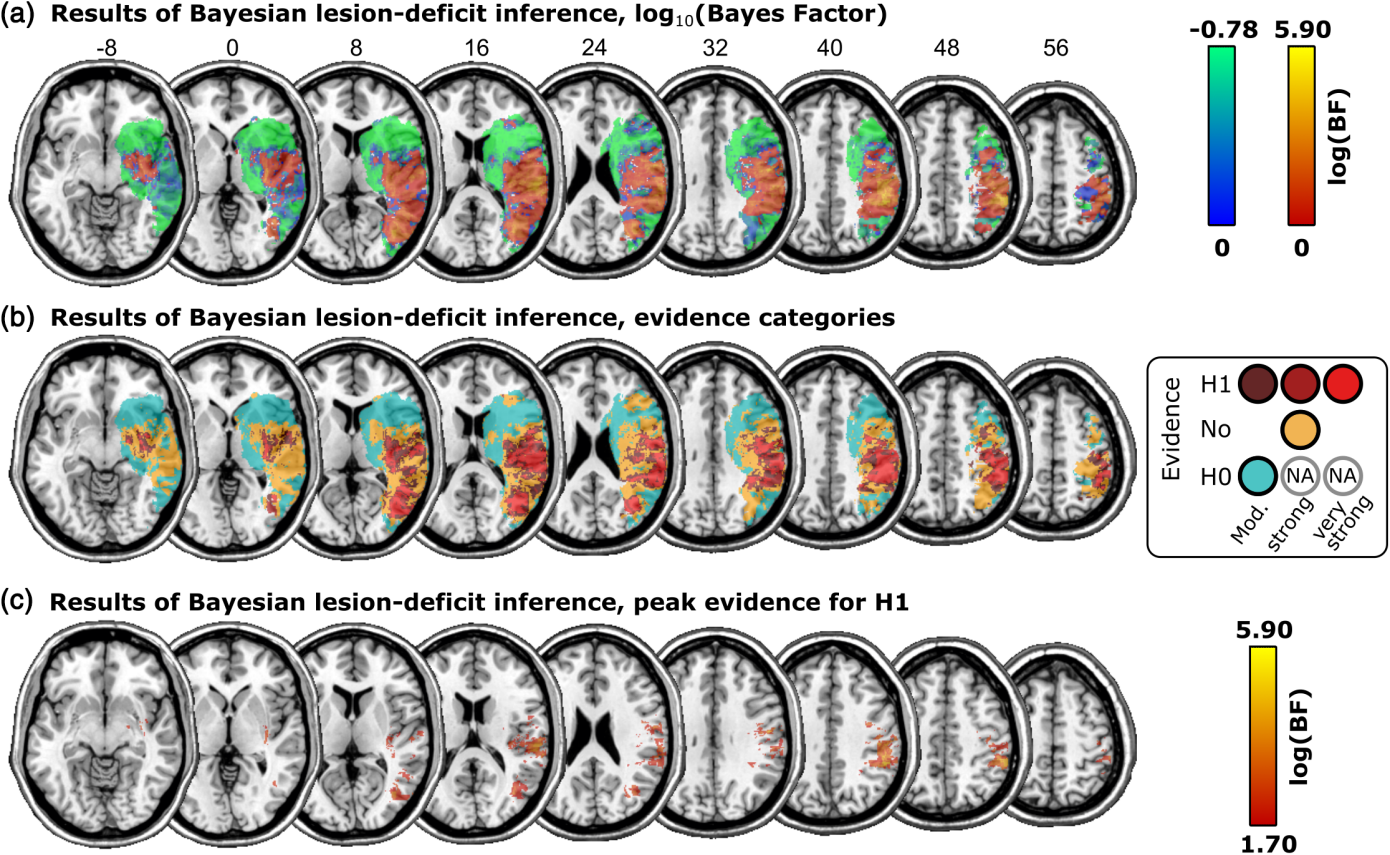
Neural substrates of visual extinction in acute stroke mapped by Bayesian lesion‐deficit inference. Extinction scores underlying the topography were controlled for spatial neglect. The figure shows (a) logarithmic Bayes factor maps in which voxels with stronger evidence for the alternative hypothesis H1 are shown in red‐yellow and voxels with stronger evidence for the null hypothesis H0 in blue‐green; (b) Bayes factor categories into evidence for H0/H1 or insufficient evidence according to established standards. (c) Areas with peak evidence for H1 (BF > 50 or log_10_(1.70)) for better comparability with the results of the multivariate analysis.

**FIGURE 4:**
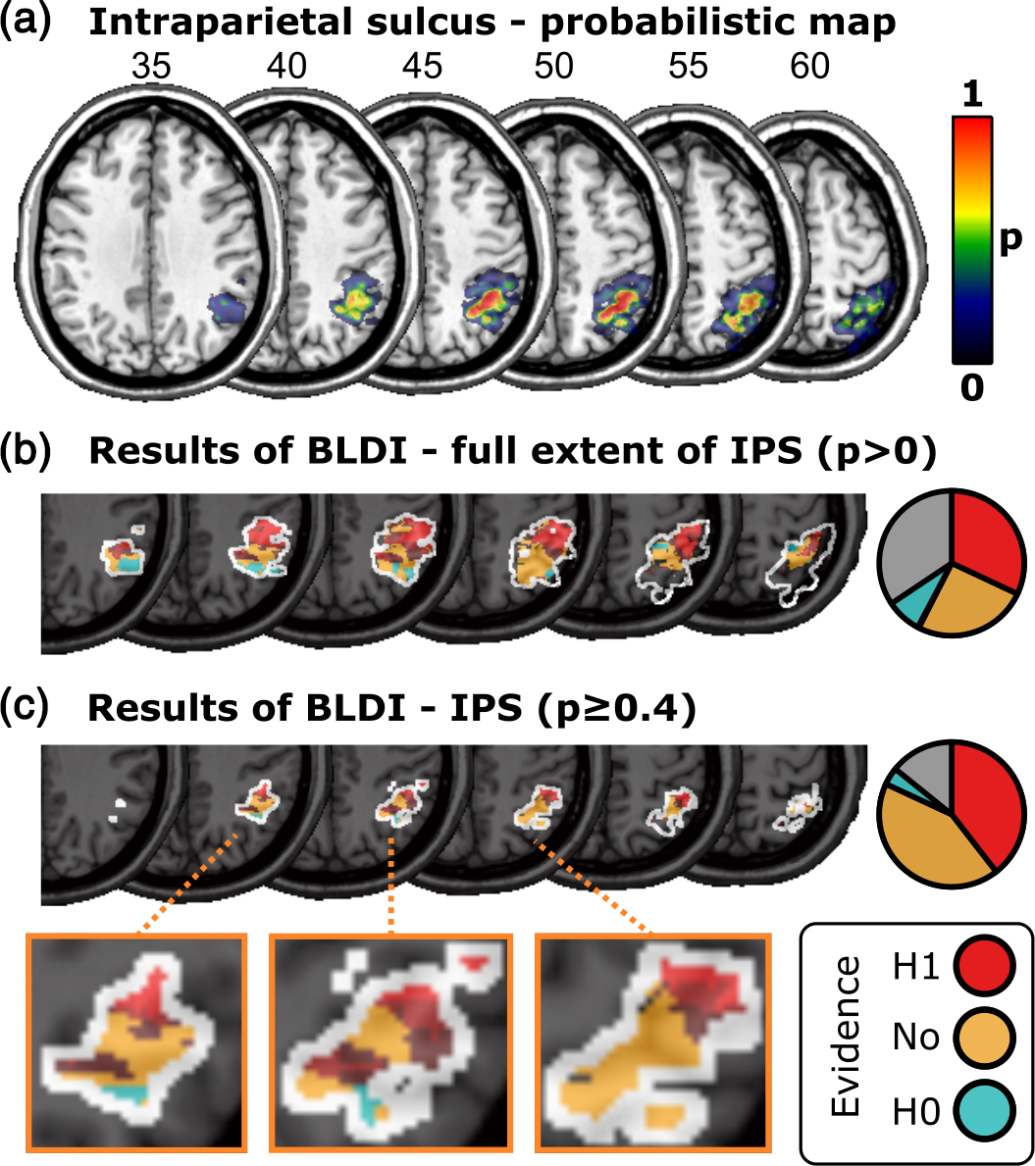
Results of Bayesian lesion‐deficit inference (BLDI) in the intraparietal sulcus (IPS). (a) Probabilistic map of the IPS as obtained from the SPM Anatomy Toolbox. (b) Bayes factors within the maximum extent map (*p* > 0) of the IPS. (c) Bayes factors within the core area (*p* ≥ .4) of the IPS.

The Bayesian analysis (see Figure 3) found areas with up to extreme evidence for an association between brain lesions and visual extinction (maximum Bayes factor ≈796,000), as well as areas with up to moderate evidence for the null hypothesis (minimum Bayes factor = 0.166). As expected for such a liberal statistical method (see Sperber et al., 2023), the areas associated with visual extinction were more wide‐spread than in the multivariate SVR‐LSM reported above. BLDI is statistically very liberal and susceptible to overestimating the neural anatomy associated with a deficit (Sperber et al., 2023). Thus, for better comparability of the Bayesian and the multivariate analyses, we focused our interpretation of the results on the peak Bayes factors (see Figure 3), at a threshold similar to that of the multivariate analysis which included a correction for multiple comparisons. This revealed a resemblance of the results of the Bayesian analysis to the results of the multivariate SVR‐LSM, with peak Bayes factors in clusters around the TPJ. An additional focus of the Bayesian analysis was the potential role of more superiorly located parietal areas, specifically the IPS. Indeed, a cluster with evidence for an association between brain lesions and visual extinction reached far into more superior parts of the parietal cortex (see Figure 3). The additional comparison with maps of the IPS (see Figure 4) suggested that visual extinction was associated with large parts of the IPS. For the core area of the IPS (see Figure 4c), we found evidence for H1 in 39.5% of voxels, insufficient evidence for either hypothesis in 42.2% of voxels, and evidence for H0 in 3.9% of voxels. Some voxels in the IPS were lesioned too rarely to be included in the analysis at all (14.2%). In summary, the Bayesian analysis, which is particularly suited in situations with low statistical power (Sperber et al., 2023), implicated substantial parts of the IPS in visual extinction, while also suggesting the absence of any anatomo‐clinical correlation in other parts of the IPS.

## 4 DISCUSSION

The current study examined the right hemisphere anatomy critical for normal multi‐target attention by investigating the anatomy that underlies visual extinction after right brain damage. Prior studies into the brain areas critical for multi‐target attention (Battelli et al., 2009; Hung et al., 2005; Koch et al., 2005; Pascual‐Leone et al., 1994) and its failure in extinction patients (Beume et al., 2020; Chechlacz, Rotshtein, et al., 2013; Chechlacz, Terry, et al., 2013; Hillis et al., 2006; Vossel et al., 2011) have produced heterogeneous findings. This heterogeneity in previous lesion analysis results may, at least in part, be due to methodological and analytical limitations leading to an underestimation of the full extent of areas of the brain critical for multi‐target attention and its failure in extinction patients. In the current study, we addressed these limitations with multivariate and Bayesian lesion analysis approaches to investigate the anatomical substrate of visual extinction in a large sample of acute right hemisphere stroke patients. This allowed us to obtain a more complete picture of the functional area or network associated with visual extinction.

Our results suggest that damage in a network of areas in and around the right TPJ underlies visual extinction. This result is in line with the results from lesion and malperfusion subtraction studies in acute patients (Karnath et al., 2003; Ticini et al., 2010), as well as the results of a univariate statistical lesion analysis study in non‐acute patients by Chechlacz, Rotshtein, et al. (2013) and Chechlacz, Terry, et al. (2013) (Analysis 1). This result is also in line with the results from several studies in neurologically healthy participants that suggest a role for the TPJ in multi‐target attention (Beume et al., 2015; Dugué et al., 2018; Meister et al., 2006). Of particular interest here is a study by Dugué et al. (2018), who identified three TPJ subregions that responded to bilateral visual stimulation whose location closely matches the location of the three clusters in and around the TPJ that we identified.

Additionally, the results from the Bayesian lesion analysis suggest that damage to the right IPS is also associated with visual extinction. This is in line with the results from both transient neurodisruption (Battelli et al., 2009; Cazzoli et al., 2009; Hilgetag et al., 2001; Hung et al., 2005; Koch et al., 2005; Pascual‐Leone et al., 1994) and neuroimaging studies in neurologically healthy participants (Çiçek et al., 2007; de Haan et al., 2015; Emrich et al., 2011; Geng et al., 2006; Gillebert et al., 2012; Mitchell & Cusack, 2008), and so resolves the seemingly conflicting findings from previous studies. Our results suggest that the reason for these seemingly conflicting findings in previous studies may be that lesion studies generally have low statistical power in these more superiorly located areas. Lesions in stroke patients most commonly affect the vasculatory territory of the middle cerebral artery (Caplan et al., 1986), which frequently includes areas in the temporal lobe and inferior parietal cortex, but rarely includes more superiorly located areas such as the IPS (Caviness et al., 2002; Phan et al., 2005; Sperber & Karnath, 2016; Stoeckel et al., 2007). Lesion analyses tend to have low statistical power to detect associations between voxel status and the deficit of interest in these less frequently damaged areas. Our Bayesian lesion analysis, however, which is better‐suited in situations of low statistical power (Sperber et al., 2023), allowed us to detect the contribution of the IPS to multi‐target attention and its failure in extinction patients.

In this context, it remains, however, puzzling that most neuroimaging studies in healthy participants implicate the IPS in multi‐target attention and its failure in extinction patients, but have failed to find evidence for a role of the TPJ (Çiçek et al., 2007; de Haan et al., 2015; Geng et al., 2006; Gillebert et al., 2012; Praß & de Haan, 2019). A key challenge underlying this is that it remains unclear what precise cognitive role the TPJ plays in multi‐target attention.

In single‐target environments, the TPJ, as part of a right‐lateralised ventral stimulus‐driven attention network, has been associated with the stimulus‐driven reorienting of attention towards unexpected behaviourally relevant stimuli presented outside of the current focus of attention (Corbetta et al., 2008; Corbetta & Shulman, 2002), or, more generally and domain‐aspecific, with the stimulus‐driven “contextual updating” of internal models of the behavioural context to allow the construction of appropriate expectations and responses following new sensory information (Geng & Vossel, 2013). Less, however, is known about the role of TPJ in multi‐target environments. The general, implicit assumption has been that the TPJ plays a very similar role in single‐ and multi‐target environments (see discussion sections in Chechlacz, Rotshtein, et al., 2013; Karnath et al., 2003; Meister et al., 2006; Ticini et al., 2010). However, a study by de Haan et al. (2015) suggests that the part of the TPJ that preferentially responds to unexpected over expected behaviourally relevant stimuli shows no preference for bilateral over unilateral stimulus presentation conditions. Moreover, the view of the TPJ as an area associated with the detection of unexpected behaviourally relevant stimuli or contextual updating of internal models of the behavioural context does not fully explain how damage to the TPJ can selectively impair multi‐target attention in the way seen in extinction patients.

A slightly different view posits that the TPJ is associated with the attentional selection/visual short‐term memory (VSTM) encoding of new sensory input, particularly in multi‐target situations. A highly influential model of selective attention views attentional selection as identical to VSTM encoding (Bundesen, 1990, 1998). As VSTM capacity is limited (Cowan, 2001), this may result in an interaction between VSTM maintenance and attentional selection/VSTM encoding to prevent the disruption of VSTM maintenance by new sensory information, particularly in multi‐target situations where VSTM capacity limits have been reached. Studies have shown that whereas IPS activity increases with higher VSTM maintenance demands, TPJ activity decreases (Todd et al., 2005). Moreover, this decrease of TPJ activity during higher VSTM maintenance demands has been linked to attentional selection deficits (Todd et al., 2005) that increase as a function of increased demands on attentional selection in multi‐target environments (Emrich et al., 2011), as well as better VSTM maintenance task performance (Anticevic et al., 2010). Overall, this pattern suggests that TPJ deactivation during higher VSTM maintenance demands helps prevent the disruption of VSTM maintenance by new sensory information (Shulman et al., 2007; Todd et al., 2005). In this view, the TPJ is associated with the attentional selection/VSTM encoding of new sensory input, particularly in multi‐target situations, a function that sometimes may have to be suppressed to support goal‐directed behaviour.

One considerable problem with this explanation, however, is that overall there is little support for the idea that activity in the TPJ increases as a function of increasing attentional selection/VSTM encoding demands. As mentioned above, apart from the single study by Beume et al. (2015), increasing attentional selection/VSTM encoding demands typically results not in an increase of activity in the TPJ, but instead in an increase of activity in the IPS (Çiçek et al., 2007; de Haan et al., 2015; Geng et al., 2006; Gillebert et al., 2012; Praß & de Haan, 2019). Indeed, some studies have suggested that the TPJ specifically responds to “target singletons” (Gillebert et al., 2012), and transient disruption of the part of the TPJ deactivated during increased VSTM maintenance demands does not appear to modulate attentional selection/VSTM encoding (Praß & de Haan, 2019). This poses the conundrum that whereas TPJ *deactivation* (whether transiently, because of increased VSTM maintenance demands, or permanently, because of brain damage in extinction patients) impairs attentional selection/VSTM encoding, particularly in multi‐target environments, TPJ *activation* does not seem reliably correlated with attentional selection/VSTM encoding demands. One possible solution to this conundrum is the proposal that the TPJ does not directly contribute to multi‐target attention, but that functional damage to the TPJ simply results in remote dysfunction in the IPS (e.g., diaschisis‐like effects, Feeney & Baron, 1986), similar to what has been proposed for spatial neglect (Corbetta & Shulman, 2011). In this view, the IPS is associated with both VSTM maintenance and attentional selection/VSTM encoding, and the critical site for the interaction between VSTM maintenance and attentional selection/VSTM encoding in multi‐target situations where VSTM capacity limits have been reached. This would fit well with the literature that implicates the IPS in both VSTM maintenance (Emrich et al., 2011; Mitchell & Cusack, 2008; Todd & Marois, 2004) and attentional selection/VSTM encoding (Çiçek et al., 2007; de Haan et al., 2015; Emrich et al., 2011; Geng et al., 2006; Gillebert et al., 2012; Mitchell & Cusack, 2008; Praß & de Haan, 2019). Moreover, there are suggestions in the literature that structural damage to the TPJ is associated with functional impairments in the IPS (He et al., 2007; Umarova et al., 2011). However, to the best of our knowledge, no study so far has been conducted to assess the relation between this remote dysfunction of the IPS following damage to the TPJ and multi‐target attention. Indeed, the study by Umarova et al. (2011) suggests that this remote dysfunction at the IPS may represent a general consequence of right hemispheric brain damage independent of the presence or absence of attentional deficits.

Some researchers have instead suggested that the TPJ is associated with the integration of information across space and time (Davis et al., 2009; Hanayik et al., 2019; Husain & Rorden, 2003), as part of a “when” pathway located between the dorsal “where/how” and the ventral “what” pathways (Agosta et al., 2017; Battelli et al., 2007). The ability to integrate information across space and time is particularly crucial in multi‐target environments, where multiple objects temporally overlap. As such, it has been suggested that a failure of this ability critically underlies extinction (Hanayik et al., 2019). Indeed, several studies have shown that extinction patients are impaired in temporal order judgment tasks (Baylis et al., 2002; Rorden et al., 1997). However, as these studies did not assess brain‐damaged patients without extinction, it is difficult to dissociate between general consequences of brain damage and deficits that are specific to extinction patients. Moreover, given that attention influences temporal perception (Hikosaka et al., 1993; Stelmach & Herdman, 1991), it is difficult to determine whether such deficits of temporal perception are the cause or a consequence of extinction. Other studies have found that patients with damage to the parietal lobe, and the TPJ in particular, are abnormally slow to process visual information (Duncan et al., 1999; Peers et al., 2005). These studies, however, did not assess whether these deficits were associated with extinction. The only study that did attempt to assess the link between impaired temporal processing and extinction (Habekost & Rostrup, 2006) only assessed patients with minor or no clinical signs of extinction. As such, their result that impaired temporal processing correlated moderately with extinction severity is difficult to interpret.

Taken together, our results suggest that both the TPJ and the IPS are critically associated with visual extinction following right hemisphere brain damage. This resolves the seemingly conflicting previous findings where lesion studies have tended to implicate the TPJ in multi‐target attention and its failure in extinction patients, whereas studies in neurologically healthy participants have tended to implicate the IPS. However, the question remains as to why studies in neurologically healthy participants so rarely implicate the TPJ in multi‐target attention and its failure in extinction patients. One reason for this may be the lack of clarity on the precise cognitive role of the TPJ in multi‐target attention and its failure in extinction patients. Over the years, various roles have been postulated for the TPJ, and these different views are far from mutually exclusive. Further research is needed to clarify the precise role of the TPJ in multi‐target attention and its failure in extinction patients.

## CONFLICT OF INTEREST STATEMENT

The authors have no conflicts of interest to declare.

## Data Availability

Online materials are publicly available at OSF under a CCBY license: https://doi.org/10.17605/OSF.IO/NVP54. These include descriptive and statistical topographies and extended demographic data. The clinical datasets analysed in the current study are not publicly available due to the data protection agreement approved by the local ethics committee.
